# Observational evaluation of children’s respirator donning ability in Indonesia and Nepal

**DOI:** 10.1038/s41598-026-64861-6

**Published:** 2026-08-04

**Authors:** Judith Covey, Sarah Nila, Chandika Shrestha, Hastin Melur Maharti, Claire J. Horwell, Kusum Shahi, Dicky C. Pelupessy, Meghnath Dhimal, Rachel L. Kendal

**Affiliations:** 1 Department of Psychology, Durham University, Durham, UK; 2 Department of Anthropology, Durham University, Durham, UK; 3 Faculty of Psychology, Universitas Indonesia, Jakarta, Indonesia; 4 Nepal Health Research Council, Kathmandu, Nepal; 5 Institute of Hazard, Risk & Resilience, Department of Earth Sciences, Durham University, Durham, UK

**Keywords:** Children, KN95 respirators, Air pollution, Respirator donning, Protective equipment, Diseases, Health care, Medical research, Risk factors

## Abstract

Certified respirators, like KN95 N95, FFP2 and KF94, can offer strong protection against airborne particulates, but their effectiveness relies on proper fit and donning. This study evaluated how well children aged 6–13 years in Kathmandu, Nepal, and Bandung City, Indonesia, could independently don two child-marketed KN95-style respirators without guidance. Among 348 participants, only 21.8% met all seven essential donning criteria, though 79.6% successfully completed at least five steps. While this suggests a promising baseline, the most frequent mistake, improperly shaping the nose clip, could potentially compromise respirator effectiveness. Older children and girls generally performed better, and prior experience with KN95-style respirators was linked to greater confidence and improved technique. Respirator design also played a role in donning success. These findings reveal a critical gap between general mask-wearing familiarity and the specific skills needed for effective respirator use. The study highlights the urgent need for child-friendly respirator designs and accessible training materials, especially in regions where air pollution poses serious health risks.

## Introduction

Fine particulate matter (PM_2.5_) is a major contributor to air pollution and has been identified as a leading global risk factor for reduced life expectancy. Its health burden surpasses that of tobacco use, infectious diseases, and violence^1^. Globally, children are especially vulnerable, with over 90% of those under 15 years exposed to PM_2.5_ concentrations exceeding the World Health Organization’s air quality guidelines^2^. Early-life exposure has been linked to impaired lung development, increased asthma risk, and potential neurodevelopmental delays^3^. Beyond respiratory and cardiovascular effects, air pollution is also associated with reproductive, neurological, and immune system dysfunctions^4^. Encouragingly, improvements in air quality have been shown to yield measurable gains in childhood lung function, underscoring the benefits of effective pollution control^5^. However, such improvements often require long-term structural and policy changes, making interim protective strategies essential - particularly for vulnerable populations like children.

One short-term protective strategy is the use of respiratory protection, which can reduce inhalation of airborne particulates. Among these, certified respirators - such as N95/99 (US), FFP2/3 (EU/UK), KN95 (China), and KF94 (Korea) - offer the highest filtration efficiency in both laboratory and real-world conditions^6–8^. However, their effectiveness depends heavily on achieving a proper seal to the face. Poor fit can significantly increase inward leakage and reduce protection - particularly in children, whose facial dimensions vary widely^9,10^. To fully benefit from respirator use, children must wear them correctly and consistently.

The COVID-19 pandemic familiarized many children with mask-wearing due to widespread mandates aimed at curbing virus transmission^11,12^. While this experience may have increased general familiarity with face coverings, it remains unclear whether it equipped children with the specific skills needed to use certified respirators effectively – particularly for protection against particulate air pollution, which presents different challenges than viral transmission.

Even among adults, proper mask-wearing technique cannot be assumed. Several studies have documented errors in mask use across both public and occupational settings. In public settings, incorrect mask usage has been observed across diverse populations, with several recurring types of errors. One issue is incomplete coverage, such as leaving the nose exposed or wearing the mask below the chin, documented in Canada and the United States^13^, Japan^14^, Ethiopia^15^, and Pakistan^16^. Another problem is loose-fitting masks that fail to provide adequate protection, as observed in Hong Kong^17^. In Singapore, only a minority of the public were able to correctly don an N95 respirator without guidance, with common errors including incorrect strap placement, visible gaps between the respirator and the skin, and improper adjustment of the nose clip^18^. Similar challenges have been reported in post-disaster New Orleans, where improper seal checks and incorrect strap placement were frequently observed^19^.

In occupational settings, where mask proficiency is especially critical, similar issues have been documented among healthcare workers despite expectations of training and expertise^20–22^. Common lapses in Sudanese healthcare workers included failing to adjust the mask at the nose bridge and not securing the mask properly^20^. Improper mask positioning (i.e., failing to ensure that the mask extended from the bridge of the nose to under the chin) has also been observed amongst healthcare workers in Nigeria^21^, and Egypt^22^.

These findings underscore the variability in adult mask-wearing proficiency and the importance of training. They also raise important questions about how well children, who may have less cognitive and physical ability than adults, can use certified respirators effectively - especially given that they tend to learn by observing adult behavior^23^. To explore these questions, the present study investigates children’s ability to use certified respirators marketed for their age group correctly. Children were observed donning certified respirators provided by researchers without receiving prior instructions. The study examines factors influencing correct respirator use, common mistakes made by children, and characteristics associated with incorrect usage. Understanding these elements is vital for designing targeted interventions to improve respirator-wearing practices among children and enhance their protection from the harmful effects of particulate air pollution.

This study forms part of the Factors Affecting Childhood Exposures to Urban Particulates (FACE-UP) project, a multidisciplinary initiative aimed at understanding and reducing children’s exposure to urban air pollution^24^. The project focused on children living in two highly polluted cities - Kathmandu, Nepal and Bandung City, Indonesia – and sought to develop, practical, evidence-based solutions. Both cities are densely populated with over 15,000 people per km^2^^25,26^ and, in recent years, have recorded average annual PM_2.5_ concentrations that exceed WHO guidelines by a factor of 7 to 10^27^. They also share similar topography. Both cities sit in bowl-shaped basins surrounded by mountains, which contributes to the trapping of air pollutants. By assessing how well children can use certified respirators in these contexts, this study supports the goal of informing future public health interventions.

## Methods

### Participants

The research was conducted from 12 December 2023 until 26 January 2024 using a protocol approved by the ethics committees of the Department of Psychology, Durham University (REF: PSYCH-2023-08-15T10_48_49-lpdh17), Nepal Health Research Council (NHRC) Nepal (REF: 615/2023), and the Faculty of Psychology, Universitas Indonesia (REF: 308/FPsi.Komite Etik/PDP.04.00/2023). All research was performed in accordance with relevant guidelines/regulations Written informed consent was obtained from all parents/caregivers, alongside children’s assent.

Children were recruited from three different schools in each country, chosen to represent local variation in socioeconomic background. Prior to the study, local research team members held orientation meetings with teachers to facilitate participant recruitment. We used stratified random sampling to select 190 children aged 7 to 12 years from each country, based on a final target of 170. The number selected from each school was proportionate to the total number of children in that age range at each school. Children who had taken part in any of the previous work packages of the FACE-UP project were excluded. When performing random selection, if more than one child from a household was chosen, only the first randomly selected child was recruited. Each selected child received a consent form and information sheet to take home to their parents, ensuring that parents were fully informed about the study and had the opportunity to decide whether to allow their children to participate. In addition to the consent forms and information sheets, several other documents were provided, including a simplified information sheet and assent form (required in Nepal) for the child and a demographic questionnaire.

Parents were informed about the types of activities that they and their children would participate in, including an interview with the children, an observation of the child donning a respirator, and a survey to be completed by parents. In this paper, we report only the findings of the observation task. Parents were also asked for permission to record video and photograph the children’s faces while donning the respirators.

### Interview with children

One-to-one interviews with the children were conducted in school by research assistants (RAs) to assess children’s past mask-wearing behavior. Amongst other questions (which are reported separately), the children were asked: (1) whether they had worn a mask in the past to protect themselves from air pollution, viruses, or other reasons (options: Yes, many times; Yes, several times; Yes, a few times; No, never; Don’t know); (2) whether they had worn a KN95 respirator in the past (options: Yes, No); and (3) the type of mask most often worn in the past (options: KN95, DuckBill, Surgical, Cloth). To support accurate reporting, children were shown pictures of different mask types before being asked about their prior use. RAs recorded the children’s responses and audio-recorded the interview. Children were reminded that they could opt out at any time or omit any questions they did not wish to answer. After the interview, the respirator donning task was undertaken.

### Respirator donning task

**Fig. 1:**
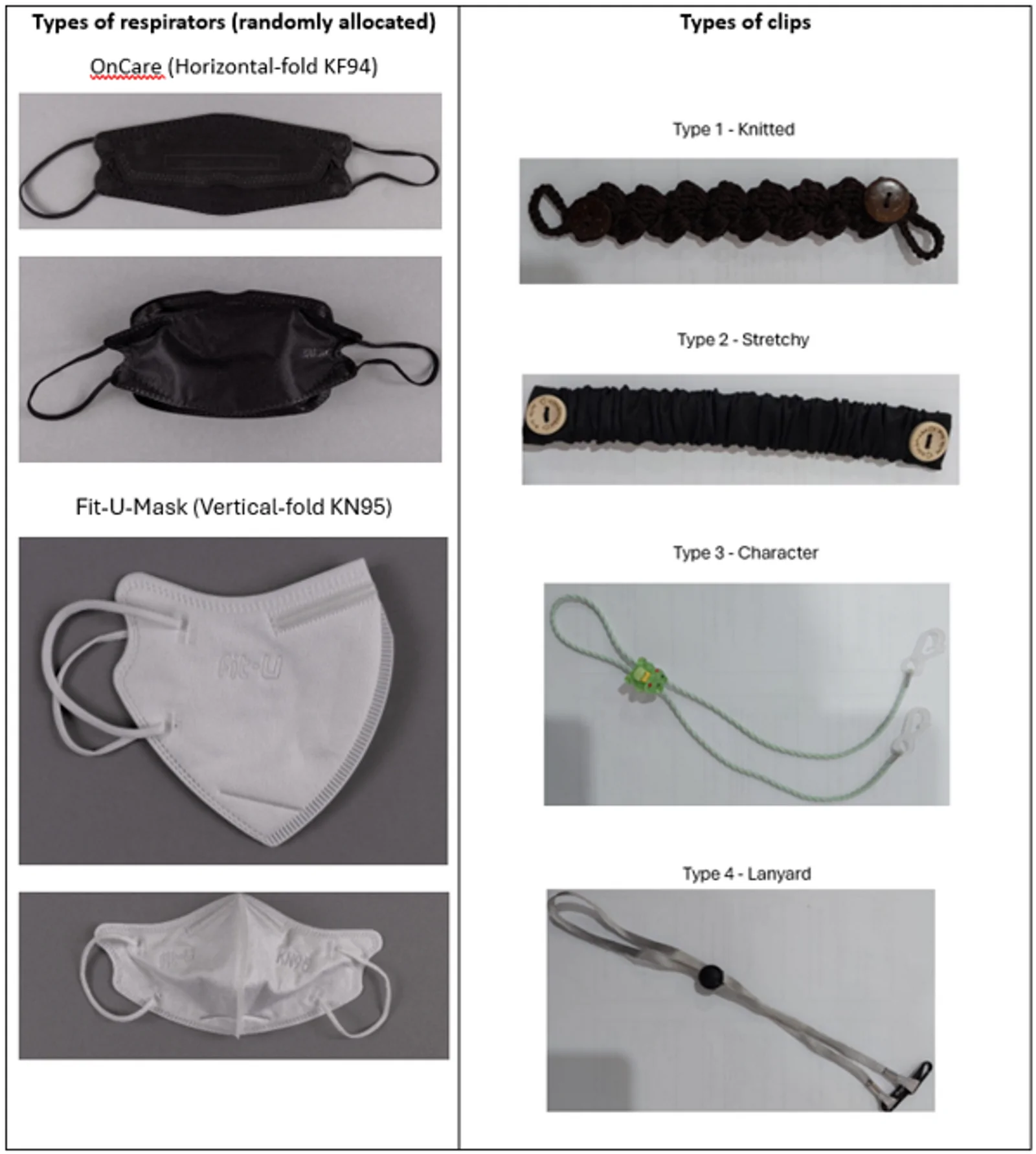
Respirators and clips used for the respirator donning task.

With this activity, we aimed to assess children’s ability to correctly don a certified respirator marketed for children. Each child was randomly assigned one of two ear loop respirators shown in Fig. 1 - either the vertical-fold Fit-U-Mask KN95, or the horizontal-fold OnCare KF94 (Fig. 1). Ear loop respirators were chosen because they are the most common type available for the public (children and adults) in Indonesia and Nepal. The alternative – with one or two elastic head straps – was extremely hard to find as they are targeted for (adult) occupational settings. Both respirators were sourced in Indonesia and had been shown in a separate study, conducted as part of the FACE-UP project, to meet the particulate penetration requirements from the US NIOSH N95 filtering facepiece respirator standard^28^.

We also provided children with four different types of clips which they could use to secure the respirator to their head (see Fig. 1). These clips are frequently used in Indonesia, particularly among women and girls who wear a hijab. The clips are attached to the ear loops of the mask and tightened around the back of the head to form a head strap. As well as tightening the respirator to the head and, thus, improving fit^9^, they can also improve comfort around the ears.

Before the task, children were informed of the procedure, including video recording and photography, using age-appropriate language and terms familiar in the local context (i.e., “mask” rather than “respirator”). Each child was given their respirator in its original folded form and asked to rate confidence in their ability to put it on correctly on a five-point scale (definitely not-0, probably not-1, maybe-2, probably yes-3, definitely yes-4). Children were then asked to don the respirator without instruction. They could choose to use a mirror and, if they had previously used a clip, they could choose to use one of the four clips provided (see Fig. 1).

The donning activity was video recorded, and photographs were taken from different angles when the child had indicated they had finished. Children then reported whether they thought the respirator fitted them well (yes-1, no/don’t know-0) and whether they would consider wearing it in the future (definitely not-0, probably not-1, maybe-2, probably yes-3, definitely yes-4). The child was also told that they could keep the respirator if they wanted to and their choice to keep it recorded (yes-1, no-0). Children were then given a small gift as a token of appreciation for their participation.

### Scoring of the respirator donning task

The scoring criteria used for the donning task are shown in Table 1. The criteria used were based on official guidance on how to correctly wear a respirator from organizations such as the US Occupational Safety and Health Administration^29^, Hong Kong Centre for Health Protection^30^, UK Health and Safety Executive^31^, respirator manufacturers such as 3M^32^, and existing public guidance on the donning of respiratory protection during volcanic eruptions^33^. Seven of the criteria were scored on all the children because they did not depend on how they secured the respirator or whether they were wearing glasses: not wearing the respirator inside out (C1); not wearing the respirator upside down - i.e., nose clip should be on top (C2); respirator should be worn fully unfolded (C3), so that it fully covered the child’s nose and mouth (C4), and under their chin (C5); the nose clip should be formed/shaped by using both hands to mold the nose clip to the shape of the nose (C6a) whilst avoiding pinching the nose clip with one hand (C6b). The other criteria were scored on sub-groups of the children because they depended on how they secured the respirator to their head, whether they were wearing glasses, or the type of respirator they were assigned. A respirator should be securely attached either by putting the ear loops behind the ears (C7a) and tightening with a knot if needed (C7b); or using a clip that is attached to the ear loops and fixed around the back of the head (C8). For children who wore glasses they should not interfere with the fit of the respirator (C9). Additional criteria were specific to the type of respirator. If the child was asked to don the Fit-U-Mask vertical-fold respirator (which is supplied folded in half (see Fig. 1)) we scored whether the child flattened the nose clip before putting it on (C10). For the OnCare horizontal-fold respirator, where the nose clip starts as a flat strip like a surgical mask, we scored whether they bent the nose clip before putting it on (C11).

**Table 1:** Criteria from the respirator donning task and the frequency (%) of participants that scored ‘yes’ on each criterion.

		Kappa/ Gwet’s AC1 (% agreement)	Full sample		OnCare (Horizontal-fold)		Fit-U-Mask (Vertical-fold)	Nepalese sample		Indonesian sample	
Criteria scored on all children		N = 40	N = 348		N = 172		N = 176	N = 185		N = 163	
C1. Respirator not inside out		1.00 (100%)	342 (98.3%)		167 (97.1%)		175 (99.4%)	180 (97.3%)		162 (99.4%)	
C2. Respirator not upside down		0.84 (98%)	303 (87.1%)		147 (85.5%)		156 (88.6%)	159 (85.9%)		144 (88.3%)	
C3a. Child unfolded the respirator before putting it on		1.00 (100%)	309 (88.8%)		135 (78.5%)		174 (98.9%)	159 (85.9%)		150 (92.0%)	
C3b. Respirator worn fully unfolded		0.60 (88%)	308 (88.5%)		135 (78.5%)		173 (98.3%)	159 (85.9%)		149 (91.4%)	
C4. Respirator covered the child’s nose and mouth		1.00 (100%)	343 (98.6%)		170 (98.8%)		173 (98.3%)	183 (98.9%)		160 (98.2%)	
C5. Respirator covered under the child’s chin		0.68 (93%)	311 (89.4%)		140 (81.4%)		171 (97.2%)	162 (87.6%)		149 (91.4%)	
C6. Child made adjustments to nose clip after they put the respirator on		0.89 (93%)	141 (40.5%)		91 (52.9%)		51 (29.0%)	87 (47.0%)		54 (33.1%)	
a. Formed/ molded to the nose with two hands		–	83 (23.9%)		50 (29.1%)		33 (18.8%)	45 (24.3%)		38 (23.3%)	
b. Pinched with one hand		–	51 (14.7%)		37 (21.5%)		14 (8.0%)	41 (22.2%)		10 (6.1%)	
Other – formed/ molded to shape of nose with one hand		–	2 (0.6%)		2 (1.2%)		0 (0%)	0 (0%)		2 (1.2%)	
Other – pressed on bridge of nose only		–	5 (1.4%)		1 (0.06%)		4 (2.3%)	1 (0.5%)		4 (2.4%)	
Criteria specific to using ear-loops		N = 40	N = 265		N = 127		N = 138	N = 183		N = 82	
C7a. Ear-loops fully behind ears		0.88^a^ (90%)	228 (86.0%)		110 (86.6%)		118 (85.5%)	157 (85.8%)		71 (86.6%)	
C7b. Ear-loops tightened		1.00 (100%)	6 (2.3%)		5 (2.9%)		1 (0.6%)	6 (3.3%)		0 (0%)	
No – but ear-loops not loose		–	232 (87.5%)		111 (64.5%)		121 (68.8%)	161 (88.0%)		71 (86.6%)	
No – ear-loops looked loose		–	11 (4.2%)		5 (2.9%)		6 (3.4%)	10 (5.5%)		1 (1.2%)	
Unclear		–	17 (6.4%)		6 (3.5%)		11 (8.0%)	6 (3.3%)		11 (12.9%)	
Criteria specific to using a clip		N = 40	N = 83		N = 45		N = 38	N = 2		N = 81	
C8. Clip used correctly (if appropriate tightening the lanyard button)		0.92^a^ (93%)	79 (95.2%)		43 (95.6%)		36 (94.7%)	2 (100%)		77 (95.1%)	
Criteria specific to children wearing glasses		N = 13	N = 13		N = 8		N = 5	N = 8		N = 5	
C9. Glasses not interfering with fit of respirator		1.00 (100%)	12 (92.3%)		7 (87.5%)		5 (100%)	7 (87.5%)		5 (100%)	
Criteria specific to Fit-U-Mask respirator		N = 20	n/a		n/a		N = 176	N = 92		N = 84	
C10. Child flattened/ took bend out of nose clip before putting respirator on		1.00 (100%)	n/a		n/a		3 (1.7%)	2 (2.2%)		1 (1.2%)	
Criteria specific to OnCare respirator		N = 20	n/a		N = 172		n/a	N = 93		N = 79	
C11. Child bent the nose clip before putting respirator on		0.88 (95%)	n/a		31 (18.0%)		n/a	28 (30.1%)		3 (3.8%)	

^a^Although the percent agreement was high for these criteria (≥ 90% agreement), the Kappa statistics were relatively low (i.e., ≤ 0.46). This can be attributed to the fact that the marginal distributions (i.e., how often each rater used each category) were very skewed. This increases the chance of agreement by coincidence and consequently lowers the Kappa value. Because Kappa can underestimate reliability in such situations, we also calculated Gwet’s AC1 for C7a and C8 using irrCAC package in R. Both statistics indicated very strong agreement (C7a AC1 = 0.88, 95% CI [0.75, 1.00], *p* < .001; C8 AC1 = 0.92, 95% CI [0.81, 1.00]). Taken together, these results show that although Kappa was unstable due to prevalence effects, AC1 provides a more reliable estimate of inter-rater agreement for these data.

It should be noted that C10 and C11 were not part of the original scoring system but were added later to provide insight into whether children adjusted the nose clip *before* donning the respirator. This distinction is important, as C6 only captured adjustments made *after* the respirator had been put on.

All videos were scored by C.S. and H.M. for C1-C9, whilst J.C. and S.N. scored all the videos for C10 and C11. These pairs of researchers had previously piloted the scoring systems. In each case, they independently scored the same random sample of 40 observations on each criterion. Discrepancies between raters were identified and used to refine the scoring criteria. Inter-rater reliability of the final scoring criteria was then formally assessed using a sub-sample of randomly selected videos, scored independently by either C.S. and H.M. (C1-C9) or J.C. and S.N. (C10 and C11). Agreement was quantified by calculating percentage agreement along with Cohen’s Kappa or Gwet’s AC1 values (see Table 1)^34^.

### Statistical analysis

The demographic information provided by the children’s parents/caregivers and scores from the observation task were analyzed in SPSS 29 using descriptive statistics (i.e., means, medians, percentages and frequencies). Structural equation modelling using SPSS AMOS 29 examined whether scores could be predicted by country, age, gender, caregiver education level, or the type of respirator assigned (OnCare or Fit-U-Mask), and whether the effects of these variables were mediated by variables that were also potentially related to the criterion such as whether the child had frequently worn masks in the past, whether the child reported having worn a KN95-style respirator in the past, how confident the child was in their ability to put the respirator on correctly; and whether the child chose to use a mirror when donning. Univariate analysis was run on the criterion for each variable and variables were retained if the lower and upper bounds of the 80% credible interval did not include zero. This approach allowed retention of potentially important variables that might not reach conventional significance but could contribute meaningfully to the multivariate structural model^35^. Bayesian estimation was used for the path analysis because the dependent variables were dichotomous or ordinal measures^36^, and 95% credible intervals for the lower and upper bounds of each estimate were obtained.

## Results

A total of 348 children participated in the observation task, including 185 from Nepal and 163 from Indonesia. The slightly lower participation rate in Indonesia was due to time constraints and participant availability. Two Indonesian children were recruited outside the intended age range (6–13 years instead of 7–12 years); this was identified during data analysis and approved for inclusion by the ethics committees. The mean age of participants was 9.73 years, 52.3% were girls, and the median carer education level corresponded to completion of secondary/higher secondary education (Table 2). Nepalese children were significantly older than the Indonesian sample (10.1 vs. 9.29 years; t = 4.49, *p* < .001), and their caregivers had lower education levels (Mann-Whitney U = 11490, Z = -3.92, *p* < .001). The higher percent of girls in the Indonesian sample (55.2% vs. 49.7%) was not statistically significant (X^2^(1) = 1.05, *p* = .307). Most children had prior experience of wearing masks, with over three-quarters reporting frequent use. Many reported having worn KN95-style respirators in the past (35.9% in Nepal and 53.7% in Indonesia), although surgical masks were more common in Nepal and DuckBill masks (a type of vertical-fold mask where the filter sits only at the front rather than comprising the entire mask material) in Indonesia.

**Table 2:** Children’s demographic characteristics, past mask wearing behavior, and responses before and after the observation task.

N	Full sample	Nepalese sample	Indonesian sample
	348	185	163
Demographic characteristics			
Age – Mean years (SD)	9.73 (1.70)	10.1 (1.64)	9.29 (1.66)
Gender - N (%) girls	182 (52.3%)	92 (49.7%)	90 (55.2%)
^a^Caregivers’ education level			
Median (25, 75 percentiles)	5.00 (4.50, 6.00)	5.00 (3.625, 6.00)	5.00 (5.00, 6.50)
Missing	12	9	3
Past mask wearing behavior			
Never worn mask for air pollution protection, virus protection or other reasons N (%)	4 (1.1%)	1 (0.5%)	3 (1.8%)
Worn mask many times for air pollution protection, virus protection or other reasons N (%)	250 (71.8%)	159 (85.9%)	91 (55.8%)
Worn KN95 respirator in past N (%)	155 (44.5%)	68 (36.8%)	87 (53.4%)
Type of mask worn most often in past N (%)			
KN95-style	35 (10.1%)	16 (8.6%)	19 (11.7%)
DuckBill	124 (35.6%)	4 (2.2%)	120 (73.6%)
Surgical	154 (44.3%)	139 (75.1%)	15 (9.2%)
Cloth	34 (9.8%)	26 (14.1%)	8 (4.9%)
Missing	1 (0.3%)	0	1
Responses before observation task			
Was the child confident in their ability to put the respirator on correctly? N (%) definitely/ probably yes	302 (86.8%)	162 (87.6%)	140 (85.9%)
Did the child want to use a clip to secure the respirator? N (%) yes	102 (29.3%)	11 (5.9%)	91 (55.8%)
Did the child want to use a mirror to put the respirator on? N (%) yes	211 (60.6%)	101 (54.6%)	110 (67.5%)
Responses after observation task			
Did the child think the respirator fitted them well? N (%) yes	292 (83.9%)	159 (85.9%)	133 (81.6%)
Would the child consider wearing the respirator they put on in the future? N (%) definitely/ probably yes	279 (80.2%)	169 (91.4%)	110 (67.5%)
Did the child keep the respirator for future use? N (%) yes	278 (79.9%)	176 (95.1%)	102 (62.6%)

^a^The carer’s and (if applicable) their spouse’s education level was obtained on a scale from 1 = No formal schooling, 2 = Some primary [grades 1–5], 3 = Completed primary [grades 6–8], 4 = Completed middle school [grades 6–8], 5 = Completed secondary/ higher secondary school [grades 9–12], 6 = Completed post-secondary/ vocational training but not degree level, 7 = Completed University Bachelor’s degree or other degree-level qualification, 8 = Completed University Masters or Postgraduate degree. In the 282 cases where information from both the carer and their spouse was provided there was a strong and significant Spearman’s correlation between the education levels (r_s_ = 0.719, *p* < .001). For these cases, the mean education level was obtained.

Before the observation task, 86.8% of children reported being probably or definitely confident in their ability to don the respirator correctly. After the task, 83.9% felt it fitted well, 80.2% would consider wearing it in future, and 79.9% chose to keep it. Notably none of these responses were significantly related to the type of respirator the child was given, but the Indonesian children were significantly less likely than the Nepalese children to consider wearing the respirator in the future (67.5% in Indonesia, 91.4% in Nepal: X^2^(1) = 31.1, *p* < .001), or choose to keep it (62.6% in Indonesia, 95.1% in Nepal: X^2^(1) = 57.2, *p* < .001).

As shown in Table 1, most children performed well on the respirator donning task, with over 85% correctly completing the basic criteria such as correct orientation (C2), full unfolding (C3), and facial coverage (C4, C5). The main exception was nose clip adjustment: only 23.9% molded the nose clip with both hands. Consequently, only 21.8% of children met all seven criteria, although 79.6% met at least five criteria. Notably, there was no association between the children’s level of confidence in their ability to don the respirator correctly and their performance (r_s_ = 0.030, *p* = .580), although there was a small positive association between donning performance and perceived fit (r_s_ = 0.215, *p* < .001).

Additional criteria were assessed for relevant sub-samples. As shown in Table 1, only 1.7% flattened the nose clip of the Fit-U-Mask *before* use, while 18.0% pre-bent the nose clip on the OnCare respirator. Among those who did not flatten the Fit-U-Mask nose clip, approximately one quarter made an adjustment after donning, either by molding the nose clip with two hands (17.9%) or pinching it with one hand (8.1%). However, 70.5% made no adjustment to the nose clip at any stage. In contrast, among those who did not pre-bend the OnCare nose clip, nearly half adjusted it after donning by molding with two hands (27.9%) or pinching with one hand (17.9%). Nevertheless, 43.0% made no adjustment at all, despite the clip being flat when removed from the packaging. Criteria also varied by how the respirator was secured. Of the 83 children who used ear-loop clips, 95.2% did so correctly (C8). Among the 265 children who relied on ear loops alone, 86.0% positioned them fully behind the ears (C7a), and most did not require additional tightening (C7b).

Table 1 also shows the percentage of participants scoring ‘yes’ for each criterion split by country and (where applicable) respirator type (OnCare or Fit-U-Mask). Children using the horizontal-fold OnCare respirator were less likely than those using the vertical-fold Fit‑U‑Mask to fully unfold the respirator (C3a: 78.5% vs. 98.9%), wear it fully unfolded (C3b: 78.5% vs. 98.3%), and ensure it covered the chin (C5: 81.4% vs. 97.2%). However, they were more likely to adjust the nose clip, either by molding it with two hands (C6a: 29.1% vs. 19.8%) or pinching it with one hand (C6b: 21.5% vs. 8.0%). Compared with Indonesian children, Nepalese children were less likely to wear the respirator fully unfolded (C3b: 85.9% vs. 92.0%) but more likely to bend the nose clip of the OnCare respirator before donning (C11: 30.1% vs. 3.8%). These patterns were consistent with the univariate modelling results shown in Table 3.

Due to lack of variance in the scores for some criteria, it should be noted that this modelling was only undertaken on the criteria where at least 10% of the children scored either ‘yes’ or ‘no’ and the sample size was large enough for the minimum of 10 cases per estimated parameter (i.e., C13, C15, C8, C19, and C25). And although more than 10% of the children scored ‘no’ on C3b (88.5% “yes”) it was not included in the modelling because scores on this criterion were almost perfectly correlated with C3a (ф = .986). Only one of the children who had fully unfolded the respirator before they put it on did not wear it fully unfolded on their face. They completely failed to put the respirator on.

In addition to the significant univariate effects of respirator type on the children’s ability to wear the respirator fully unfolded (C3b), wear it so that it was covering their chin (C5), and mold the nose clip with two hands (C6b), the analysis showed that boys were less likely than girls to wear the respirator fully unfolded (C3b), not upside down (C2), or covering their chin (C5). Older children were also more likely to wear the respirator the correct way up (C2) and mold the nose clip with two hands (C6a).

**Fig. 2:**
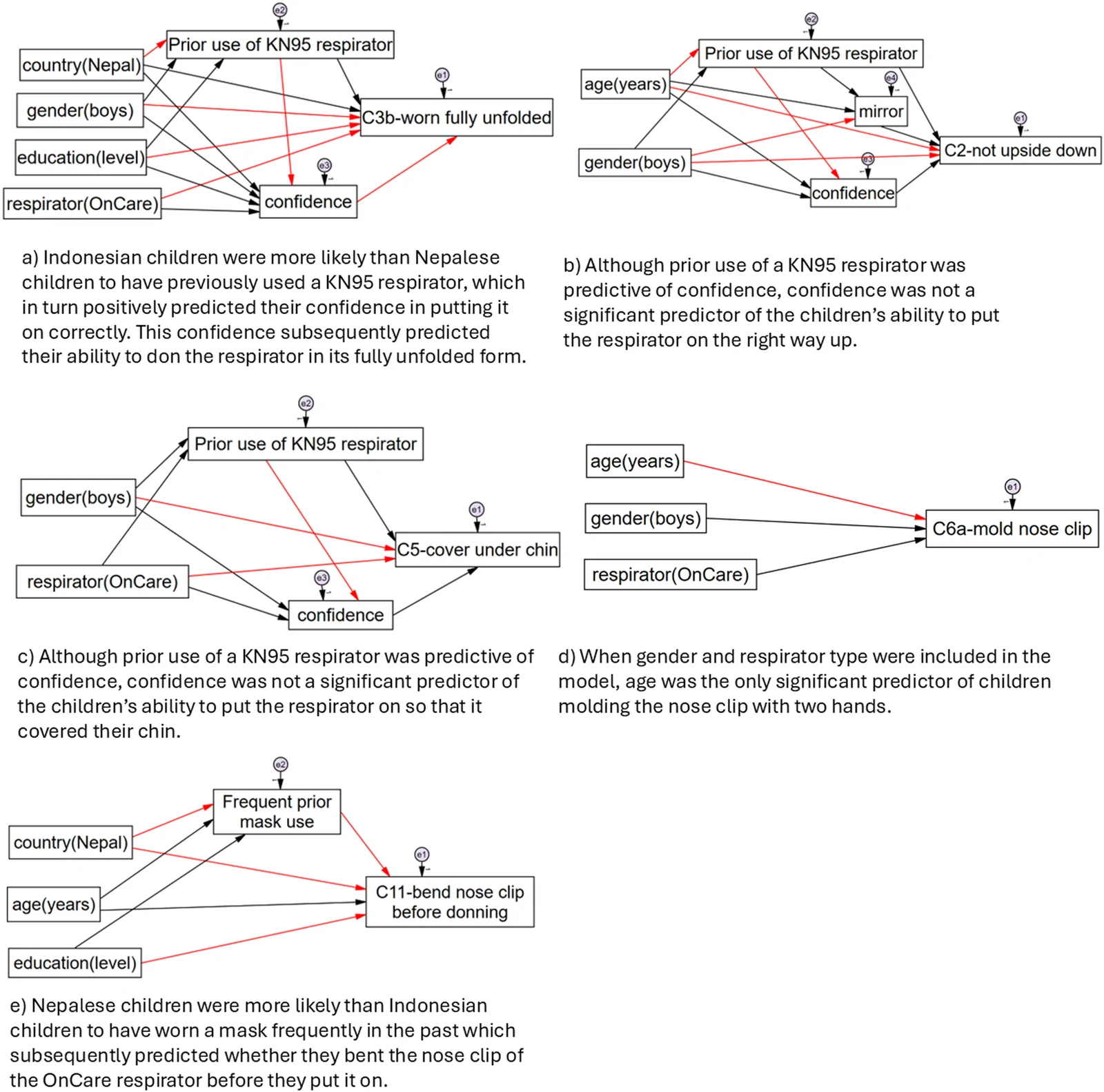
(**a**–**e**) Path diagrams for the structural modelling. Significant path coefficients are shown in red.

The structural modelling included all the exogenous and endogenous variables shown in italics in Table 3 that had a coefficient with an 80% credible interval that did not include zero. The potential mediators tested as endogenous variables included: having worn a mask many times in the past (C11), having worn a KN95-style respirator in the past (C3b, C2, C5), confidence in their ability to put the respirator on correctly (C3b, C2, C5), and choosing to use a mirror to put the respirator on (C2). No potential mediators were identified for C6a (nose clip molded with two hands). All the models exceeded the minimum sample size requirement of 10 times the number of parameters considered in the analysis and posterior predictive p values were close to 0.50 (0.47 − 0.51) indicating the models were plausible^36^. For each estimate, the upper and lower endpoints of a 95% credible interval (95% CI) were obtained and coefficients for which this interval did not include zero are shown in red and marked with an asterisk (*) in Fig. 2a and e. For these estimates there is therefore a 95% or higher probability that the true estimate is not zero.

**Table 3:** Univariate analyses.

b coefficient^a^	C3b− worn fully unfolded	C2− not upside down	C6a−nose clip molded two hands^b^	C5−cover under chin	C11−bent nose clip (OnCare Kids respirator)
Exogenous variables					
Respirator type (OnCare = 1, Fit−U−Mask = 0)	−0.198**	*−*0.032	0.109*	−0.158**	n/a
Country (Nepal = 1, Indonesia = 0)	*−0.055* ^*¥*^	*−*0.024	0.008	*−*0.039	0.264**
Age (years)	0.010	0.043**	0.034*	< 0.001	0.043*
Gender (boys = 1, girls = 0)	−0.102**	−0.099**	*−0.075* ^*¥*^	−0.085*	*−*0.019
Caregiver education level^c^	*−0.019* ^*¥*^	−0.011	−0.007	−0.014	−0.085**
Endogenous variables					
Frequent prior mask use (yes = 1, no = 0)	0.018	−0.009	0.045	−0.033	0.210**
Prior use of KN95−style respirator (yes = 1, no = 0)	*0.056* ^*¥*^	0.081*	−0.024	0.075*	0.049
Confidence in ability to don respirator correctly (scale)	0.050**	*0.028* ^*¥*^	0.016	0.035*	0.032
Mirror used during donning (yes = 1, no = 0)	0.039	*0.051* ^*¥*^	0.044	0.029	−0.040

^¥^80% credible interval, †90% credible interval, *95% credible interval, **99% credible interval.

^a^For categorical variables (e.g., respirator type, country, gender), the unstandardized coefficients (b) indicate whether the probability of ‘yes’ scores were higher (positive coefficient) or lower (negative coefficient) in the category coded 1 relative to 0. For example, a coefficient of -0.198 for Respirator type tells us that the children using the OnCare respirator were on average 19.8 percentage points less likely to wear the respirator fully unfolded than the children using the Fit-U-Mask. For continuous or ordinal variables (i.e., age, caregiver education level, and confidence) the unstandardized coefficients (b) tell us how much the probability of ‘yes’ scores increase (positive coefficient) or decrease (negative coefficient) with each one unit increase in the predictor. For example, a coefficient of 0.034 for Age tells us that for each additional year of age the probability of molding the nose clip with two hands increases by 3.4% points on average. Variables with coefficients shown in *italics* were included in the structural modelling.

^b^The significance of the predictors in the univariate or multivariate structural modelling for this criterion was not affected by including two cases where the child molded the nose clip with one hand.

^c^Note that 12 of the caregivers did not provide information about their or their spouse’s education level. The coefficients shown here are based on median imputation of missing values. Sensitivity analysis confirmed that the significance of this predictor was the same before median imputation.

As shown in Fig. 2a, children from Indonesia and older children were more likely to report that they had used a KN95-type respirator in the past (country(Nepal) -> prior use of KN95 respirator: *b* = -0.157, 95% CI [-0.266, -0.049]; age(years) -> prior use of KN95 respirator: *b* = 0.036, 95% CI [0.004, 0.067]. And girls were more likely to use a mirror than boys (gender(boys) -> mirror: *b* = -0.146, 95% CI [-0.248, -0.042]). Figure 2e shows that children from Nepal were more likely to report they had worn a mask frequently in the past for any reason (country(Nepal) - > frequent prior mask use: *b* = 0.316, 95% CI [0.179, 0.455]). However, there were the only two credible connections between these variables and the respirator donning criteria.

First country had an indirect effect on achieving criterion 3b (mask fully unfolded), operating sequentially through prior KN95 respirator use and confidence (indirect effect = -0.0017 95% CI [-0.0047, -0.0001]). As shown in Fig. 2a, Indonesian children were more likely than Nepalese children to have previously used a KN95 respirator (country(Nepal) -> prior use of KN95 respirator: *b* = -0.157, 95% CI [-0.266, -0.049]). Prior use of a KN95 respirator, was in turn, associated with greater confidence in donning the respirator correctly (prior use of KN95 respirator -> confidence: *b* = 0.262, 95% CI [0.067, 0.461]), and greater confidence subsequently predicted correct achievement of criterion 3b, wearing the respirator in its fully unfolded form (confidence -> C3b-worn fully unfolded: *b* = 0.041, 95% CI [0.006, 0.075]).

Second country had an indirect effect on achieving criterion 11 (bent nose clip), operating through frequent prior mask use (indirect effect = 0.041 95% CI [0.005, 0.087]). As shown in Fig. 2e, Nepalese children were more likely than Indonesian children to report frequent prior mask use (country(Nepal) -> frequent prior mask use: *b* = 0.316, 95% CI [0.179, 0.455]). Frequent prior mask use, in turn, predicted achievement of criterion 11, bending the nose clip of the OnCare respirator before donning it (frequent prior mask use -> C11-bent nose clip: *b* = 0.129, 95% CI [0.013, 0.251]).

## Discussion

This study provides one of the first large-scale, real-world assessments of children’s ability to independently don certified respirators in high-pollution urban environments. While only a minority of children (21.8%) met all seven core donning criteria, the majority (79.6%) successfully completed at least five. Most children were able to correctly position the respirator over their nose and mouth, wear it right-side up, and ensure it was fully unfolded and covered under the chin. However, correct molding of the nose clip was uncommon with only 23.9% using the recommended two-handed technique. These findings suggest that, although children are generally capable of performing many aspects of respirator donning, one of the key technical actions essential for achieving a proper seal remains a challenge without guidance.

Molding the nose clip with both hands is critical for shaping it to the contours of the nose, removing any gaps, especially at the apex above the bridge of the nose. One-handed pinching can create a pointed bend that compromises the seal. The low prevalence of correct adjustment is therefore of concern and mirrors patterns previously observed in adult populations^13,18,19^. This step may be especially difficult for younger children due to limited dexterity or lack of understanding of the nose clip’s existence or purpose, compounded by the likelihood that this is also not modelled well by adults they may observe.

Demographic and contextual factors were significantly associated with variation in performance, but these should be interpreted cautiously. Older children tended to perform better, consistent with developmental improvements in fine motor skill development^37,38^. For instance, 3-year-old girls have been found to outperform boys in fine motor activities such as drawing, cutting, and manipulating small objects^37^. Although these sex differences diminish with age^39^, girls aged 7–12 attending a primary school in Malaysia were shown to exhibit greater attention to detail than boys^40^, which may contribute to more precise and complete execution of multi-step procedures like respirator donning. These differences therefore reflect general developmental variation rather than inherent differences in capability and would therefore be expected to diminish over time.

Similarly, differences observed between the Nepalese and Indonesian children are best understood as reflecting contextual factors such as prior exposure, mask availability, and local practices. For example, Nepalese children were more likely to engage in preparatory behaviors (e.g., bending the nose clip of the OnCare respirator), possibly reflecting greater familiarity with masks with flat nose clips, such as surgical masks. Indonesian children, in contrast, reported greater prior use of KN95-style respirators. These findings highlight how experience and context can shape behavior, rather than indicating intrinsic differences between populations. With appropriate training, children in both settings would be expected to achieve similar levels of performance.

Consistent with this, prior experience with KN95-style respirators was associated with greater confidence; although, this confidence translated into improved performance on only one criterion - wearing the respirator fully unfolded. For other key aspects of correct donning, such as ensuring the respirator was not worn upside down, properly molding the nose clip, and covering the chin, confidence alone was not a reliable predictor. This disconnect between perceived and actual ability echoes findings from adult populations^18^: confidence alone is not a reliable indicator of correct technique, reinforcing the importance of explicit instruction.

Respirator design also played a key role in donning success. Children using the vertical-fold Fit-U-Mask were more likely to wear the respirator fully unfolded and covering the chin, while those using the horizontal-fold OnCare model were more likely to adjust the nose clip. These differences suggest that certain design features may either facilitate or hinder correct use. For example, the Fit-U-Mask’s pre-bent nose clip (due to vertical-fold masks being supplied folded) may have reduced the perceived need for manual adjustment when, in reality, the nose clip is ‘over bent’ and must be flattened to prevent a gap at the bridge of the nose compared to the OnCare’s flat clip which required more obvious and active manipulation. Similarly, the (vertical) Fit-U-Mask, though supplied folded, cannot physically be donned without unfolding it, whereas the (horizontal) OnCare respirator’s presentation in the packaging (with upper and lower flaps folded onto the central part of the respirator) can be (incorrectly) donned without fully unfolding it. These findings underscore the importance of designing child-specific respirators that are intuitive to use and accommodate children’s physical and cognitive capabilities. Here, children’s ability to perceive the affordances of the respirator, or the possible ways of interacting with or using it^40^ could be enhanced with ‘user-centered design’. Signifiers^41^ could help children understand the affordances of respirator features, such as arrows indicating that the flaps of horizontal respirators should be opened.

A key strength of this study lies in its ecological validity: children were observed donning respirators without instruction, simulating real-world conditions in which they might be expected to use such devices independently. However, this approach may underestimate children’s potential ability with even minimal explicit guidance or the opportunity to observe and copy the actions of experts. Indeed, children have a strong propensity to learn by observing others^42,43^ especially adults^44^ and can discern and preferentially learn from experts by 7–8 years^45^. In addition, the cross-sectional design provides only a snapshot of children’s abilities, without capturing how donning skills might improve with training or repeated exposure. The study was also limited to two urban centers, Kathmandu and Bandung which, while offering valuable cross-cultural insights, may not be representative of broader regional or global populations. Importantly, while the assessment captured observable indicators of correct donning (e.g., mouth and nose coverage and positioning), it did not directly evaluate the quality of the face seal or overall fit using formal fit-testing methods. As face seal integrity is a key determinant of respirator effectiveness, this represents a limitation. However, fit testing was conducted in another work package of the FACE-UP project^9^. However, in that study, children were assisted by researchers to don the respirator correctly, including opening the respirator fully and molding the nose clip. Consequently, the findings provide an estimate of the level of fit and exposure reduction achievable when respirators are fitted under near-optimal conditions. Real-world protection depends however not only on the intrinsic fit capability of the respirator but also on children’s ability to don and adjust it correctly. The present study therefore complements the fit-testing data by examining the extent to which children can independently achieve the conditions necessary for effective respiratory protection.

Despite these limitations, the findings have important implications for public health policy and practice. As air pollution continues to pose a serious threat to child health in many urban areas, certified respirators may offer a valuable interim protective measure. However, their effectiveness depends not only on their filtration efficiency but also on their fit and correct use. The research presented in this study has already contributed to the development of educational resources, including an instructional video and comic-strip infographic for children (involving characters children identify as ‘experts’ in this context) and an information booklet for parents and caregivers, available via UNICEF’s Children’s Environmental Health Collaborative knowledge library^46^. These materials provide clear, age- and education-level- appropriate guidance on how to correctly don face masks and respirators to optimize protection. A forthcoming paper evaluates the potential for these products to enhance respirator donning skills.

Looking ahead, future interventions should prioritize improvements in child-specific respirator design starting with global consensus manufacturing standards and selection and usage guidance^47^, integrate donning instruction into school curricula, and ensure that caregivers and educators are equipped to support children in using respirators effectively. Tailored training tools - such as visual aids, interactive demonstrations, and hands-on practice - may help bridge the gap between general mask familiarity and the technical skills required for effective respiratory protection.

## Data Availability

Data supporting the results reported in this article can be found from the following link: https://doi.org/10.17605/OSF.IO/PTKSX.
